# Prevalence of acne and its impact on quality of life and practices regarding self-treatment among medical students

**DOI:** 10.1038/s41598-024-55094-6

**Published:** 2024-02-22

**Authors:** Duaa Dabash, Haya Salahat, Sally Awawdeh, Fadi Hamadani, Husam Khraim, Amer A. Koni, Sa’ed H. Zyoud

**Affiliations:** 1 Department of Medicine, College of Medicine and Health Sciences, An-Najah National University, Nablus, 44839 Palestine; 2 Division of Clinical Pharmacy, Department of Hematology and Oncology, An-Najah National University Hospital, Nablus, 44839 Palestine; 3 Department of Clinical and Community Pharmacy, College of Medicine and Health Sciences, An-Najah National University, Nablus, 44839 Palestine; 4 Poison Control and Drug Information Center (PCDIC), College of Medicine and Health Sciences, An-Najah National University, Nablus, 44839 Palestine; 5 Clinical Research Center, An-Najah National University Hospital, Nablus, 44839 Palestine

**Keywords:** Acne, Prevalence, Severity, Acne disability, Medical students, Self-treatment, Palestine, Health care, Medical research

## Abstract

Acne vulgaris is one of the most common skin diseases worldwide and causes great distress to patients. In addition, most acne patients suffer from low self-esteem and social withdrawal. This study aimed to assess the prevalence of acne and its impact on quality of life among medical students. It also evaluates the patterns of self-treatment use. The study population consisted of all medical students from An-Najah National University (ANU) and the hospital. The questionnaire consists of three parts, and the first part consists of questions regarding demographic information. The second part consisted of questions to measure the severity of acne using the acne severity scale as well as the Cardiff Disability Index, which assesses the quality of life concerning acne in medical students. Finally, the third part consisted of questions exploring and assessing acne self-treatment. The mean age of our study sample was 21.3 ± 1.9 years, with a female predominance of 72.3%. The prevalence of acne among medical students was 80.9%, and 36.6% practiced self-medication. Acne was strongly associated with female sex (*p* < 0.001) and skin type (*p* = 0.024). Regarding diet, dairy consumption (*p* = 0.007), sweets (*p* < 0.001), chocolate (*p* < 0.001), and oily food (*p* = 0.006) were all significantly associated with acne. Skin type was strongly associated with the severity of acne (*p* < 0.001) and the Cardiff acne disability index (*p* = 0.016). Gender (*p* = 0.039) was also associated with Cardiff acne disability. A significant correlation was found between the severity of acne and impaired quality of life. The most commonly used topical agent for self-treatment remedies was antibiotics (70.3%). The most commonly used oral agent was isotretinoin (9.4%). A total of 22.7% of the students used herbal products, while 47.7% used home remedies. Acne is prevalent among medical students, with a high percentage of students having different degrees of impairment in their daily lives. As a result, self-medication among acne sufferers is highly common. Awareness of the appropriate use of self-medication should increase among medical students.

Acne vulgaris is a common skin condition that affects people of all ages and ethnicities. It is caused by inflammation of the pilosebaceous unit, which consists of a hair follicle and a sebaceous gland. Inflammation can be triggered by a variety of factors, such as excess oil production, bacteria, hormonal imbalances, and genetics^1^. The clinical presentation of acne vulgaris can vary widely but typically includes the presence of comedones (blackheads and whiteheads), papules (small, raised bumps), pustules (pus-filled bumps), nodules (large, painful lumps), and, in severe cases, scarring. Acne can also affect different areas of the body, including the face, neck, chest, and back^2,3^.

Most acne patients suffer from low self-esteem, social withdrawal, and depression because their skin carries the stigma of the disease for the world to see and criticize daily. In some patients, acne can have detrimental effects and complications that can be avoided by educating patients about the importance of seeking help from a specialist and compliance with acne treatment. Acne is typically not an acute disease but rather a condition that constantly changes in its distribution and severity^4^. Acne treatment is necessary for many months and sometimes years^5^.

The prevalence of acne vulgaris in medical students ranged from 34.38 to 97.9%^6^. In Saudi Arabia, the incidence of acne among students of health-related science colleges is 78.5%, with 56.0% using self-medications without a prescription^7^. In Syria, the incidence of acne was 34.7%, and the face was the most common site for acne^8^. In Malaysia, the prevalence of acne was 75.8%. Compared with male students, female students had significantly impaired quality of life, and students with acne had higher rates of frequent insomnia than did those without acne^9^.

Self-medication for the treatment of acne can involve over-the-counter (OTC) medicines, herbal or traditional medicines, or other products such as dietary supplements^10^. Self-medication is a common practice in many countries, and it can be helpful when used responsibly and with appropriate knowledge and guidance^11^. In practice, it also includes the use of family members, especially in regard to the treatment of children or elderly individuals. Self-medication is an area of interest in the dermatology field. Medical students often grasp their knowledge of the pharmacology and treatment of skin diseases from their textbooks, senior medical colleagues, and students. In addition, medications from pharmaceutical sales representatives are available to physicians urging medical students to self-treat their acne.

Acne vulgaris can have a significant impact on a patient's quality of life, causing emotional distress, social anxiety, and depression. According to a previous review, there is a significant association between acne and anxiety and depression^12^. Importantly, there is also concern about suicidal thoughts among adults due to this condition, which requires clinicians to inform patients and their families that depression symptoms should be evaluated frequently in patients with acne^13,14^. Treatment options for acne include topical and oral medications, as well as lifestyle modifications and skincare practices. It is important for patients to work closely with their dermatologist to find the most effective treatment plan for their specific case of acne^15^.

The risk factors mentioned by Palestinian patients who potentiate or exacerbate acne were emotional stress, fatty food, and chocolate^16^. Another study revealed that pharmacists had inadequate knowledge of acne treatment^17^. In Palestine, no study has been conducted to determine the prevalence of acne or its effect on quality of life among the public or a specific group. Therefore, this study aimed to assess the prevalence of acne and its impact on quality of life among medical students. It also evaluates patterns of self-treatment. The results of this study could help create an awareness strategy for acne and its management.

## Methods

### Study design

Our questionnaire-based cross-sectional study was used to determine the prevalence of acne and self-treatment among medical students at An-Najah National University (ANU) in Palestine. We chose this study design because it is the most suitable and feasible way to assess the prevalence of self-treatment for acne among medical students at ANU in Palestine.

### Study setting

This study was carried out at ANU in Nablus, northern West Bank, Palestine.

### Study population

The study population consisted of medical students at ANU, including first-year through sixth-year students, with a male-to-female ratio of 1:2^18^. Therefore, basic science and clinical-level knowledge are included to provide further insight into the topic and represent the majority of the population, as students attending the university come from different cities in Palestine.

### Sample size and procedure

A convenience sample of 350 medical students was used from ANU in Nablus, West Bank, Palestine. Research coordinators contacted the university and the hospital participants and subsequently sent the questionnaires. Here, the RaoSoft sample size calculator is used to achieve a confidence interval of 95% and a standard error of 5% with a response distribution of 50%:

- We used the Raosoft sample size calculator website: https://www.raosoft.com/samplesize.html.
- Enter the following information:
- Population size: Enter the estimated size of the population you are interested in studying. If you do not know the population size, you can leave this field blank or enter an estimate.
- Margin of error: Enter 5%, which is the standard error you want to achieve.
- The confidence interval was set to 95%.
- Expected frequency: Enter 50%, which is the expected proportion of responses in the population.
- Click the "Calculate Sample Size" button.
- The calculator will provide you with the recommended sample size you need to achieve your desired confidence interval and standard error.

### Inclusion and exclusion criteria

The target population in our study was medical students from An-Najah National University and Hospital. The inclusion criteria dictate that medical students of all ages and sexes be enrolled at the university and hospital in Nablus, Palestine, from the first to the final year. The exclusion criteria dictate nonmedical students.

### Data collection instrument

A questionnaire was used to collect data from medical students; this questionnaire was developed based on previous studies^11,19^. All participants were asked to provide verbal consent to participate in the study. After agreeing to participate, they were asked to complete a three-part questionnaire. The first part consisted of demographic information, including age, sex, height, weight, and other acne-related questions. The second part consists of questions to measure the severity of acne using the acne severity scale^20^ and the Cardiff Disability Index, which assess the quality of life regarding acne in medical students^21^. Finally, the third part consisted of questions exploring and evaluating acne self-treatment, ranging from drugs to creams and natural products.

### Ethical approval and consent to participate

The *Institutional Review Board (IRB) of An-Najah National University* approved this study. The authors issued appropriate permission documents for the study. The students were free to accept or reject the invitation to participate in the study voluntarily. Verbal informed consent was obtained from each subject who agreed to participate in this study. The confidentiality of the data was ensured. The *IRB of An-Najah National University* approved only verbal informed consent. The reason for verbal informed consent is that participants were only needed for the interview and were not subjected to any harm as long as their privacy was kept confidential. The authors confirmed that all the methods were performed following the relevant guidelines and regulations.

### Statistical analysis

The study used IBM-SPSS version 21, a software program commonly used for statistical analysis, to analyze the data. The data are presented as percentages and frequencies. The Kolmogorov‒Smirnov test was used to test for normality of the variables. The Kruskal‒Wallis test and Mann‒Whitney U test were applied as appropriate. These tests were used to compare groups of continuous or ordinal data that were not normally distributed. The chi-square test was used to test for significant differences between categorical variables, which can be placed into categories or groups. The Pearson test was used to assess the correlation between two continuous variables, specifically between the acne severity index and the Cardiff acne disability score. A p-value less than 0.05 was considered statistically significant.

## Results

### Demographic data and other characteristics

The mean age of our study sample was 21.3 ± 1.9 years. Of the 350 respondents, 72.3% responded to the questionnaire, suggesting a gender preference.

Although 63.1% of the students had a normal body mass index (BMI), i.e., (18.5− < 25), 23.1% were overweight with a BMI of 25− < 30 kg/m^2^. A total of 6.6% had an obese BMI (30), and 7.1% were underweight with a low BMI (< 18.5). Among the female participants, 83.8% had normal menarche at 12–15 years of age. A total of 77.5% reported that their period was regular, and no abnormalities regarding menses were reported with respect to menses, such as heavy bleeding, bleeding at irregular intervals, or severe pain during menses. Most of our study participants (67.4%) were in the clinical stage, i.e., in the fourth, fifth, or sixth year. Regarding smoking, 86.6% of the students reported that they were nonsmokers. The predominant skin type was oily (33.1%), followed by combination skin (28.6%) and normal skin (25.7%). Medical students are under significant stress, as 49.7% of the students reported that their current mental status is stressed. A total of 50.9% of the participants reported consuming oily foods as a major part of their daily diet, while approximately 46% reported consuming dairy, sweets and chocolate. Table 1 presents the demographics and characteristics of the participants in detail.

**Table 1:** Characteristics of the participants (n = 350).

Variable	Frequency (%)
Age	
< 20	72 (20.6%)
20–23	242 (69.1%)
> 23	36 (10.3%)
BMI	
Underweight (BMI < 18.5)	25 (7.1%)
Normal Weight (BMI 18.5- < 25)	221 (63.1%)
Overweight (BMI 25- < 30)	81 (23.1%)
Obesity (≥ 30)	23 (6.6%)
Menarche *	
< 12	24 (9.5%)
12–15	212 (83.8%)
> 15	17 (6.7%)
Sleeping hours	
4–6	112 (32.0%)
7–9	210 (60.0%)
10–12	26 (7.4%)
> 12	2 (0.6%)
Study year	
First year	42 (12.0%)
Second year	38 (10.9)
Third year	34 (9.7%)
Fourth year	61 (17.4%)
Fifth year	90 (25.7%)
Sixth year	85 (24.3%)
Gender	
Male	97 (27.7%)
Female	253 (72.3%)
Chronic diseases	
Yes***	15 (4.3%)
No	335 (95.7%)
Smoking	
Yes	47 (13.4%)
No	303 (86.6%)
Skin type	
Oily	142 (33.1%)
Other type**	208 (66.9%)
Period regularity****	
Regular	196 (77.5%)
Not regular	54 (21.3%)
Current mental and emotional status	
Stressful	173 (49.7%)
Not stressful	177 (50.3%)
Diet	
Dairy	161 (46.0%)
Sweets	164 (46.9%)
Nuts	133 (38.0%)
Chocolate	161 (46.0%)
Oily food	178 (50.9%)
Spicy food	111 (31.7%)
Frequent use of cosmetics	
Frequently used	73 (20.9%)
Not used	277 (79.1%)

*All frequencies are divided by the total number of female participants (n = 253); ** Normal, dry, combination, and sensitive; *** Diabetes and Asthma/Allergies; **** 3 female participants were missing; all frequencies were divided by the total number of female participants (n = 253);

### Sample characteristics associated with acne prevalence, severity, and disability

The prevalence was 80.9%. Gender (*p* < 0.001), smoking status (*p* = 0.017), and skin type (*p* = 0.024) were significantly associated with acne occurrence. Regarding diet, dairy products (*p* = 0.007), sweets (*p* < 0.001), chocolate (*p* < 0.001), and oily food (*p* = 0.006) were strongly associated with acne. However, the analysis did not find significant associations between acne and age, BMI, emotional status, or use of cosmetics. Table 2 presents the associations between sample characteristics and acne occurrence.

**Table 2:** Associations between sample characteristics and acne incidence (n = 350).

Variable	Frequency (%) N = 350	Students with acne Frequency (%) n = 283	Students without acne Frequency (%) n = 67	*P* value (Chi-squared test)
Age				0.072
< 20	72 (20.6%)	60 (21.2%)	12 (17.9%)	
20–23	242 (69.1%)	199 (70.3%)	43 (64.2%)	
> 23	36 (10.3%)	24 (8.5%)	12 (17.9%)	
BMI				0.206
Underweight	25 (7.1%)	22 (7.8%)	3 (4.5%)	
Normal weight	221 (63.1%)	178 (62.9%)	43 (64.2%)	
Overweight	81 (23.1%)	68 (24.0%)	13 (19.4%)	
Obesity	23 (6.6%)	15 (5.3%)	8 (11.9%)	
Sleeping hours				0.812
4–6	112 (32.0%)	89 (31.4%)	23 (34.3%)	
7–9	210 (60.0%)	172 (60.8%)	38 (56.7%)	
10–12	26 (7.4%)	20 (7.1%)	6 (9%)	
> 12	2 (0.6%)	2 (0.7%)	0 (0.0%)	
Study year				0.104
First year	42 (12.0%)	37 (13.1%)	5 (7.5%)	
Second year	38 (10.9)	29 (10.2%)	9 (13.4%)	
Third year	34 (9.7%)	32 (11.3%)	2 (3%)	
Fourth year	61 (17.4%)	44 (15.5%)	17 (25.4%)	
Fifth year	90 (25.7%)	74 (26.1%)	16 (23.9%)	
Sixth year	85 (24.3%)	67 (23.7%)	18 (26.9%)	
Gender				** < 0.001**
Male	97 (27.7%)	66 (23.3%)	31 (46.3%)	
Female	253 (72.3%)	217 (76.7%)	36 (53.7%)	
Chronic diseases				> 0.99
Yes	15 (4.3%)	12 (4.2%)	3 (4.5%)	
No	335 (95.7%)	271 (95.8%)	64 (95.5%)	
Smoking				**0.017**
Yes	47 (13.4%)	32 (11.3%)	15 (22.4%)	
No	303 (86.6%)	251 (88.7%)	52 (77.6%)	
Skin type				**0.024**
Oily	142 (33.1%)	123 (43.5%)	19 (28.4%)	
Other type	208 (66.9%)	160 (56.5%)	48 (71.6%)	
Current mental and emotional status				0.397
Stressful	173 (49.7%)	143(50.5%)	30(44.8%)	
Not stressful	177 (50.3%)	140(49.5%)	37(55.2%)	
Dairy				**0.007**
Yes	161 (46.0%)	140(49.5%)	21(31.3%)	
No	189 (54.0%)	143(50.5%)	46(68.7%)	
Sweets				** < 0.001**
Yes	164 (46.9%)	146(51.6%)	18(26.9%)	
No	186 (53.1%)	137(48.4%)	49(73.1%)	
Nuts				0.491
Yes	133 (38.0%)	110(38.9%)	23(34.3%)	
No	217 (62.0%)	173(61.1%)	44(65.7%)	
Chocolate				** < 0.001**
Yes	161 (46.0%)	144(50.9%)	17(25.4%)	
No	189 (54.0%)	139(49.1%)	50(74.6%)	
Oily food				**0.006**
Yes	178 (50.9%)	154(54.4%)	24(35.8%)	
No	172 (49.1%)	129(45.6%)	43(64.2%)	
Spicy food				0.215
Yes	111 (31.7%)	94(33.2%)	17(25.4%)	
No	239 (68.3%)	189(66.8%)	50(74.6%)	
Frequent use of cosmetics				0.184
Frequently used	73 (20.9%)	63(22.3%)	10(14.9%)	
Not used	277 (79.1%)	220(77.7%)	57(85.1%)	

Significant are in value [bold].

The mean acne severity score was 10.2 ± 7.6. The severity of acne was strongly associated with the type of oily skin type, with a *p* value < 0.001 (Table 3). The median Cardiff acne disability index for females was significantly greater than the median for males with acne. Furthermore, patients with stress had a median [Q1-Q3] of 3^1–6^, which was substantially greater than the median of unstressed individuals (Table 4).

**Table 3:** Subgrouped acne severity scores based on demographic data (n = 283).

Variable	Frequency (N%)	Acne severity score median (Q1–Q3)	*P* value
Age			0.773
< 20	60 (21.2%)	7(4–15.75)	
20–23	199 (70.3%)	9(4–15)	
> 23	24 (8.5%)	9(3.5–17.75)	
Gender			0.127
Male	66 (23.3%)	9(6–15.25)	
Female	217 (76.7%)	8(3–15.5)	
BMI			0.108
Underweight	22 (7.8%)	7(4–14.25)	
Normal weight	178 (62.9%)	8(4–14)	
Overweight	68 (24.0%)	12(4–19.75)	
Obesity	15 (5.3%)	7(3–14)	
Chronic diseases			0.654
Yes	12 (4.2%)	8.5(3.5–22.5)	
No	271 (95.8%)	8(4–15)	
Smoking			0.272
Yes	32 (11.3%)	9(6.25–17.75)	
No	251 (88.7%)	8(4–15)	
Mental status			0.066
Stressful	143 (50.5%)	9(4–18)	
Not stressful	140 (49.5%)	8(3–13)	
Frequent use of cosmetics			0.826
Yes	63 (22.3%)	8(3–18)	
No	220 (77.7%)	8.5(4–15)	
Dairy			0.987
Yes	140 (49.5%)	9(4–15.75)	
No	143 (50.5%)	8(4–15)	
Sweet			0.619
Yes	146 (51.6%)	8(4–17)	
No	137 (48.4%)	8(4–14)	
Nuts			0.425
Yes	110 (38.9%)	8(4–14)	
No	173 (61.1%)	9(4–16)	
Chocolate			0.505
Yes	144 (50.9%)	9(4–16)	
No	139 (49.1%)	8(3–15)	
Oily foods			0.561
Yes	154 (54.4%)	8.5(4–17)	
No	129 (45.6%)	8(3.5–14.5)	
Spicy			0.592
Yes	94 (33.2%)	9(4–16.25)	
No	189 (66.8%)	8(4–15)s	
Sleeping hours			0.354
4–6	89 (31.4%)	9(5–18)	
7–9	172 (60.8%)	8(4–14.75)	
10–12	20 (7.1%)	7(3.25–13.75)	
> 12	2 (0.7%)	10.5(9–12)	
Study year			0.291
1st	37 (13.1%)	6(3.5–10)	
2nd	29 (10.2%)	10(4–17)	
3rd	32 (11.3%)	6.5(3.25–15.75)	
4th	44 (15.5%)	8(4–16)	
5th	74 (26.1%)	9(7.75–14.25)	
6th	67 (23.7%)	10(3–17)	
Skin type			** < 0.001**
Oily	123 (43.5%)	11(6–18)	
Nonoily	160 (56.5%)	7(3–13)	

Significant are in value [bold].

**Table 4:** Cardiff acne disability index scale subgrouped according to demographic data (n = 283).

Variable	Frequency (N%)	Cardiff acne disability score median (Q1–Q3)	*P* value
Age			0.660
< 20	60 (21.2%)	3(1–5)	
20–23	199 (70.3%)	3(1–5)	
> 23	24 (8.5%)	2(1–4)	
Gender			**0.039**
Male	66 (23.3%)	2(0–4)	
Female	217 (76.7%)	3(1–5)	
BMI			0.923
Underweight	22 (7.8%)	3(0–5)	
Normal weight	178 (62.9%)	3(1–5)	
Overweight	68 (24%)	2(1–5)	
Obesity	15 (5.3%)	2(0–4)	
Chronic diseases			0.105
Yes	12 (4.2%)	4(2.25–8)	
No	271 (95.8%)	3(1–5)	
Smoking			0.470
Yes	32 (11.3%)	3(2–4.75)	
No	251 (88.7%)	3(1–5)	
Mental status			**0.005**
Stressful	143 (50.5%)	3(1–6)	
Not stressful	140 (49.5%)	2(1–4)	
Frequent use of cosmetics			0.098
Yes	63 (22.3%)	3(1–5)	
No	220 (77.7%)	3(1–5)	
Dairy			0.116
Yes	140 (49.5%)	3(1–5)	
No	143 (50.5%)	3(1–4)	
Sweet			0.618
Yes	146 (51.6%)	3(1–5)	
No	137 (48.4%)	3(1–4)	
Nuts			0.316
Yes	110 (38.9%)	3(1–5)	
No	173 (61.1%)	3(1–4.5)	
Chocolate			0.184
Yes	144 (50.9%)	3(1–5)	
No	139 (49.1%)	3(1–4)	
Oily foods			0.311
Yes	154 (54.4%)	3(1–5)	
No	129 (45.6%)	2(1–4.5)	
Spicy			0.092
Yes	94 (33.2%)	3(1–5)	
No	189 (66.8%)	2(1–4)	
Sleeping hours			0.731
4–6	89 (31.4%)	3(1–4)	
7–9	172 (60.8%)	2(1–5)	
10–12	20 (7.1%)	3.5(1–5)	
> 12	2 (0.7%)	3(0–6)	
Study year			0.388
1st	37 (13.1%)	3(1–5.5)	
2nd	29 (10.2%)	2(0–4)	
3rd	32 (11.3%)	3(2–6)	
4th	44 (15.5%)	3(1–4)	
5th	74 (26.1%)	3(1–6)	
6th	67 (23.7%)	3(1–4)	
**Skin type**			**0.016**
Oily	123 (43.5%)	3(2–5)	
Non-Oily	160 (56.5%)	2(.25–4)	

Significant are in value [bold].

### Severity and disability of acne

Increasing acne severity was significantly and positively associated with acne disability from acne (r = 0.175, *p* = 0.003) (Table 5).

**Table 5:** Correlations between the acne severity index and the Cardiff acne disability score.

	Cardiff acne disability score
Acne severity index	
Pearson Correlation	0.175**
Sig. (2-tailed)	**0.003**

**The correlation is significant at the 0.01 level (2-tailed).

Significant are in value [bold].

### Self-medication and acne remedies used by students

In our study, 36.6% of the students practiced self-treatment for acne, representing 45.2% of the acne students. Among those ranging from acne face washes, 26.6% were cosmetic acne cream (coal cream mask, blackhead remover cream, etc.). 28.9%, topical retinoids 10.2%, topical salicylate 17.2%, topical antibiotics 70.3%, and topical corticosteroids such as betamethasone were used by 6.3%, while herbal products, for example (chamomile, green tea, etc.) 22.7%. A total of 47.7% used home remedies such as yogurt, honey, and coffee masks. Oral acne medications, such as isotretinoin (9.4%), antibiotics (tetracycline) (7.8%) and oral contraceptives (4.7%), were minimal. Finally, 1.6% spironolactone was used. Table 6 describes the different types of acne self-medications and remedies.

**Table 6:** Self-medications and remedies (n = 128).

Type of self-treatment	Frequency
Steroids (e.g. betamethasone)	8 (6.3%)
Cosmetic creams	37 (28.9%)
Acne face wash	34 (26.6%)
Topical retinoids (adapalene)	13 (10.2%)
Topical salicylates	22 (17.2%)
Topical Antibiotic	90 (70.3%)
Herbal products (green tea, chamomile)	29 (22.7)
Home remedies (Honey, yogurt, coffee, etc.)	61 (47.7%)
Oral Isotretinoin	12 (9.4%)
Oral Antibiotic (Tetracycline)	10 (7.8%)
Oral contraceptives	6 (4.7%)
Spironolactone	2 (1.6%)

## Discussion

This group is regularly faced with clinical rotations such as those for dermatology, physicians who could influence their decisions about self-treatment of acne, and books that need to be studied for those rotations that include information on acne and acne treatment.

Acne is a well-known but still mysterious skin disease that has and still impacts people worldwide^22^. Unfortunately, acne research has not been established for Palestine or the West Bank, let alone for self-treatment of acne among medical students in Palestine. Therefore, this study was conducted to closely investigate this issue and dissect it to better understand acne, its predisposing factors, and the knowledge and attitudes of medical students toward acne and its self-treatment. Although our study revealed that the prevalence of acne was 80.9%, according to other studies around the globe, the incidence of acne ranged from 34.4% in Bangladesh^23^ to 97.9% among Saudi female medical students^24^, while two other studies in Saudi Arabia reported a prevalence of 55%^25^ and 55.5%^26^. Furthermore, the prevalence of acne was 68.1% in Malaysia^27^, 66.6% in India^28^, 62.2% in Portugal^29^, 57.8% in several European countries^30^, and 55.9% in Pakistan^31^. In China, 10.4% of students complain of moderate to severe acne^32^.

The prevalence of acne among women was greater in this study (85.8%), with a *p value* < 0.001, than in other global studies that have shown that females have a greater incidence of acne, such as a study in Egypt (28.6%)^33^ and a study in Iran (90%)^34^. This may be due to the frequent use of comedogenic makeup and the predisposition of women to acne^35^. Gender was also an important factor in determining life disability according to the Cardiff Disability Index. A study conducted in India revealed that women aged 18–25 years were severely affected by acne in terms of emotional and social impairment^36^. A previous study showed that personality type could explain the connection between acne and subjective well-being. Therefore, the study recommended strategies addressing psychological aspects, particularly stress and mood management, that may effectively enhance life satisfaction in individuals with acne^37^.

In our study, 63.1% of the students had a normal BMI (18.5- < 25), and 23.1% were overweight and had a BMI of 25- < 30 kg/m^2^. A total of 6.6% were obese (BMI ≥ 30), and 7.1% were underweight (BMI < 18.5). These results exclude the possibility that an increased BMI is correlated with an increased prevalence of acne^38^. Skin type is an essential and significant determinant of acne predisposition and development. In our study, the predominant skin type was oily (33.1%). This distribution was found to be essential for both acne and its development. Oily skin type was strongly associated with acne, with a *p* value of 0.024. The pathogenesis of acne probably explains this; it dictates overproduction as sebum clogs the pores and creates a sustainable medium for bacterial growth^39^. Sebum is produced in greater quantities in oily skin types, as supported by a previous study conducted in Seoul, Korea, on this topic^40^. An interesting finding was the association between acne severity and skin type. Our study revealed that the severity of acne was strongly associated with skin type, with a *p* value < 0.001. We could not find similar findings in further research. This is very noteworthy and should be considered when treating acne patients. Skin type was also linked to greater life disability in terms of emotional and social impairment, which is another reason to consider skin type when treating patients with acne.

Furthermore, in our study, diet was investigated, and we found that 50.9% of the oily foods consumed were associated with the development of acne (*p* = 0.006), which can be explained by the fact that increased fatty acids modulate the inflammatory response^41^, which is a determining factor in the pathogenesis of acne. A total of 46.0% of the participating students consumed dairy associated with acne (*p* = 0.007). This is likely because the bacterium of the P. acnes phenotype, which is involved in the development of acne, is found in dairy and IGF-1, which is also found in dairy and plays a role in acne pathogenesis^41,42^. Approximately 46% of the students consumed chocolate and sweets, which were associated with acne (*p* < 0.001). According to a study conducted in France, these foods have a high glycemic index and increase inflammatory cytokines^32^, which stimulate the inflammatory process of acne^43^.

Medical students face significant stress^44^, as 49.7% of the students reported their current mental state as stressed. This stressful mental status was associated with the Cardiff acne disability index, with a p value of 0.005. Although stressed students had a higher acne severity score, this difference was not significant compared to that of nonstressed students. A previous study in Saudi Arabia revealed that significant stress levels were correlated with increased severity of acne^24^.

In our study, 36.6% of the students practiced self-treatment for acne, representing 45.2% of the acne students. This finding seems similar to that of a study conducted in Pakistan that revealed that the incidence of acne self-treatment was 50.4%^45^. Another study conducted in India reported a prevalence of 59.2%^1^. Of the participants, 26.6% used acne face washes. Additionally, some participants used cosmetic acne creams, such as coal cream masks and blackhead remover creams. 28.9%, topical retinoids 10.2%, topical salicylate 17.2%, topical antibiotics 70.3%, and topical corticosteroids such as betamethasone were used by 6.3%. Topical acne treatments can explain why they seem safer than oral treatments^46^. Furthermore, topical treatments are widely used and have proven effective with minimal side effects, thus encouraging medical students to use them without prescription^47^. Herbal products (chamomile, green tea, etc.) were used by 22.7%, and home remedies such as yogurt, honey and coffee masks were used by 47.7%. These remedies have always been known to help with skin issues, so participants reported no harm from using natural remedies to treat acne or the availability of these products. Furthermore, our study reported the use of oral acne medications such as isotretinoin (9.4%) and tetracycline (7.8%), and oral contraceptives were minimally used (4.7%). Finally, 1.6% spironolactone was used. This also explains why students were more hesitant to use oral medications for fear of side effects. In addition, it was not known how to use them properly or if they were even indicated for their acne.

An impressive finding was the significant and positive correlation between acne severity and impaired quality of life. Similarities were found in previous papers^33,48,49^, while another study reported no significant correlation^50^.

### Strengths and limitations

The strengths of our study include that this is the first study to be conducted in Palestine on the self-treatment of acne and its impact on quality of life. However, our study included only medical students at ANU, one of the four universities in Palestine for medicine. Additionally, we did not exclude students with depression and anxiety. This is considered a limitation of our study. Another limitation was the cross-sectional design of the current study and the small sample size, which impeded us from generalizing the study's findings.

## Conclusions

Acne is a highly prevalent condition among medical students, and self-medication for acne is also common. The use of antibiotics without prescriptions was high, and allopathy was the most common type of self-medication used by students. Additionally, there was a significant correlation between acne severity and quality of life. Self-medication is part of self-care, so minor illnesses should be encouraged, but this should be based on detailed knowledge and restricted to over-the-counter drugs. Therefore, awareness of the appropriate use of oral medications should increase among medical students to reduce self-medication practices. In addition, the rational prescription and prevention of adverse effects of these drugs or worsening of acne should be improved.

## Data availability

The data collected and analyzed for this study are available from the corresponding author upon reasonable request. This manuscript was created as part of a Doctor of Medicine graduation project submitted to An-Najah National University. The abstract was published as part of self-archiving institutional repositories (university repository: https://repository.najah.edu/handle/20.500.11888/16056).

## Abbreviations

ANU: An-Najah National University
WHO: World Health Organization
IRB: Institutional review board
SPSS: Statistical package for social science programmes
BMI: Body mass index
IGF-1: Insulin-like growth factor-1
